# How do changes in bulk soil organic carbon content affect carbon concentrations in individual soil particle fractions?

**DOI:** 10.1038/srep27173

**Published:** 2016-06-02

**Authors:** X. M. Yang, C. F. Drury, W. D. Reynolds, J. Y. Yang

**Affiliations:** 1 Harrow Research and Development Centre, Agriculture and Agri-Food Canada, Harrow, ON, Canada N0R1G0

## Abstract

We test the common assumption that organic carbon (OC) storage occurs on sand-sized soil particles only after the OC storage capacity on silt- and clay-sized particles is saturated. Soil samples from a Brookston clay loam in Southwestern Ontario were analysed for the OC concentrations in bulk soil, and on the clay (<2 μm), silt (2–53 μm) and sand (53–2000 μm) particle size fractions. The OC concentrations in bulk soil ranged from 4.7 to 70.8 g C kg^−1^ soil. The OC concentrations on all three particle size fractions were significantly related to the OC concentration of bulk soil. However, OC concentration increased slowly toward an apparent maximum on silt and clay, but this maximum was far greater than the maximum predicted by established C sequestration models. In addition, significant increases in OC associated with sand occurred when the bulk soil OC concentration exceeded 30 g C kg^−1^, but this increase occurred when the OC concentration on silt + clay was still far below the predicted storage capacity for silt and clay fractions. Since the OC concentrations in all fractions of Brookston clay loam soil continued to increase with increasing C (bulk soil OC content) input, we concluded that the concept of OC storage capacity requires further investigation.

Soil organic carbon (OC) is typically segregated into distinct “pools” with unique characteristics and specific turnover times^1^. These pools have been used to indicate the sustainability of farming practices from a soil quality perspective^2^. The soil’s capacity to store or sequester OC appears to be determined largely by its mass proportion of fine particles^3^,^4^,^5^,^6^,^7^,^8^; and as a result, most OC storage capacity and sequestration models consider only the fine soil particulate size fraction^5^,^6^,^9^. It has been found, however, that OC storage on silt and clay can be significantly greater than model-predicted maximum levels^10^, and OC storage can vary substantially with land use, clay type, and cultivation practice^11^. It has also been suggested that OC storage may depend at least partially on specific surface area of soil clay minerals^8^, and could be as high as ~1 mg C m^−2^ (monolayer-equivalent C loading)^12^,^13^.

Although organic carbon sequestration in soil may be controlled primarily by OC associated with the “silt and clay” particles, soil OC not associated with these particles (mainly the OC in sand-sized particles) is also important because it is the main soil component which controls soil mechanical properties^2^,^14^,^15^. It has been proposed, for example, that OC accumulation on sand-sized material tends to occur only after the OC storage capacity on silt plus clay is exceeded^2^,^16^. Jagadamma and Lal^17^ further found that OC in sand and silt fractions usually decreases quickly with change in land use, while OC in the clay fraction often continues to accumulate at a reduced rate. Shrestha *et al*.^18^ noted that cultivated soils contain less clay-associated OC than forest soils, and that natural forest soils typically have more OC associated with all particle sizes. In a chronosequence of grassland restoration, He *et al*.^19^ found that increases in soil OC storage occurred primarily in the sand fraction at 0–10 cm depth, and primarily in the clay fraction below the 10 cm depth. Recently, Vogel *et al*.^20^ suggested that only the “rough edges” of clay mineral surfaces contribute to OC storage; and as a consequence, OC sequestration in soil may not be directly proportional to silt and clay content. Clearly, more study is needed to better delineate how OC in the different soil particle size-fractions changes in response to changing bulk soil OC.

In view of the above, we hypothesized that existing predictive models may underestimate OC storage capacity or “saturation levels” on soil silt and clay particle sizes. We further hypothesized that relationships among OC storage in bulk soil and on the sand, silt and clay particle size fractions could be profitably examined by considering a single soil type with a large range of OC concentrations resulting from a range of long-term soil and crop management practices. Accordingly, soil samples were collected from several long-term field trials on a Brookston clay loam, and relationships among OC storage in bulk soil and on the clay (<2 μm), silt (53–2 μm) and sand (>53 μm) particle size fractions were studied.

## Results

### OC concentrations in bulk soil and in the particle size fractions

The OC concentrations in bulk soil and in the sand, silt and clay sizes are presented in Table 1. Bulk soil OC concentrations ranged from 4.7 to 70.8 g C kg^−1^ soil with an average of 20.8 g C kg^−1^. The concentration of OC associated with particle size fractions ranged from 0.4 to 66.9 g C kg^−1^ for sand, 1.9 to 64.4 g C kg^−1^ for silt, and 6.2 to 84.0 g C kg^−1^ for clay, with the averaged values of 6.4, 21.3, and 29.9 g C kg^−1^, respectively. The concentration of OC associated with particle size fractions on a bulk soil basis ranged from 0.1 to 28.3 g C kg^−1^ bulk soil for sand, 0.6 to 20.7 g C kg^−1^ bulk soil for silt, and 2.9 to 28.9 g C kg^−1^ bulk soil for clay, with the averaged values of 1.8, 7.1 and 11.8 g C kg^−1^ bulk soil, respectively.

### Relationships between the OC concentrations on different sized particles (g C kg^−1^ particle fraction) and the OC in bulk soil (g C kg^−1^ bulk soil)

**Figure 1:**
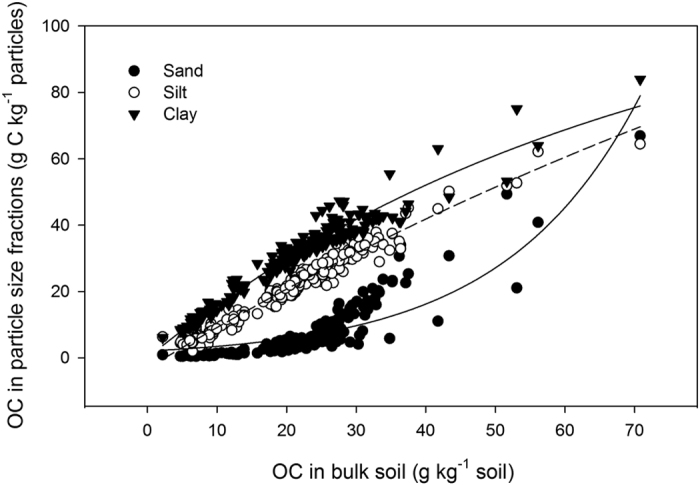
Relationships between the organic carbon (OC) concentration in soil particle size fractions and OC in bulk soil.

Empirical exponential growth models (Equations 2 and 3) fitted the OC concentration data very well with P < 0.0001, and R^2^ = 0.73, 0.96 and 0.95 for sand, silt and clay, respectively (Fig. 1, Table 2). The corresponding OC rate constants (*b* values) varied greatly from 0.051 kg sand g^−1 ^C for sand, to 0.014 kg clay g^−1 ^C for clay, to 0.006 kg silt g^−1 ^C for silt. Despite the fact that the clay rate constant was a factor of 2.3 greater than the silt rate constant, the predicted storage capacity for the clay fraction (118 g C kg^−1^ clay particle, *y*_*0*_ + *a*, Table 2) was only about half that for the silt fraction (202 g C kg^−1^ silt particle, *y*_*0*_ + *a*, Table 2). The maximum OC concentration for the silt-sized particles was predicted by the model to occur when the bulk soil organic C concentration was at ~700 g C kg^−1^ whereas the maximum OC concentration in the clay-size fraction occurred when the bulk soil C was ~250 g kg^−1^. From the perspective of increasing bulk soil carbon levels over time, the clay sized particles would become saturated with C far before the silt particles, while the OC in the sand sized particle would continue to increase as bulk soil OC increases (Fig. 1, Table 2).

### Proportions of OC in size fractions relative to OC in bulk soil

**Figure 2:**
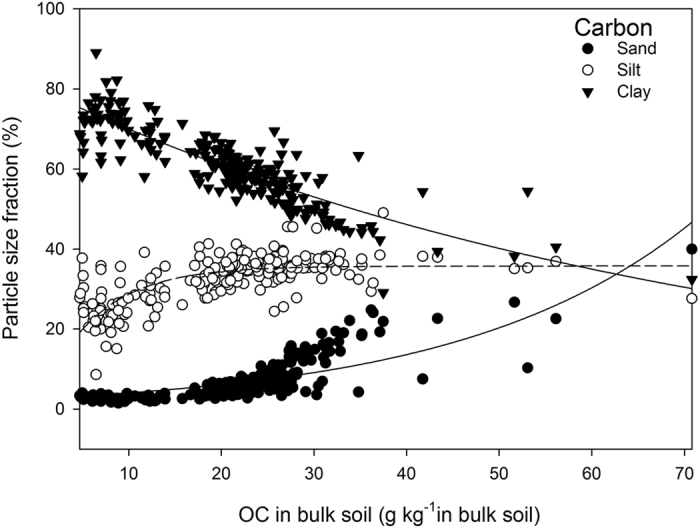
Proportional changes of organic carbon (OC) on soil particle size fractions in response to increasing bulk soil OC.

**Figure 3:**
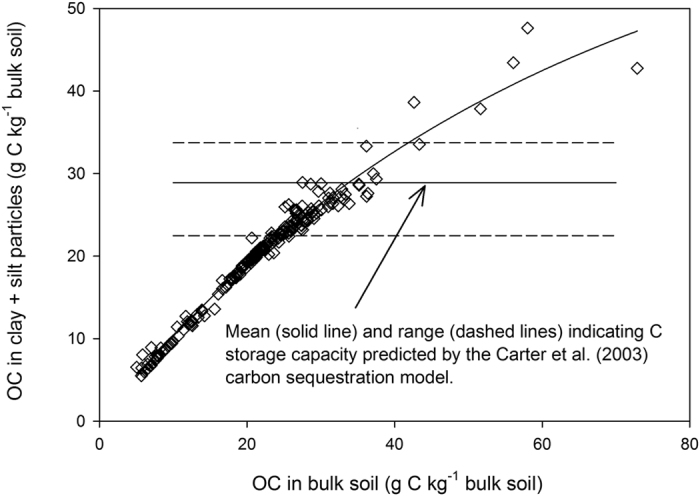
Relationship between organic carbon concentration (OC) in the silt + clay particle size fraction (y) and in bulk soil for Brookston clay loam. The 3 horizontal lines depict mean and range of OC storage capacity on silt plus clay using the Carter *et al*. (2003) carbon sequestration model.

The relative proportions of OC in the soil particle size fractions varied with OC concentration in bulk soil (Fig. 2, Table 3). When bulk soil OC concentrations were low (10 to 15 g kg^−1^ soil), over 70% of OC was associated with clay, less than 5% with sand, and the remainder with silt. Increasing bulk soil OC resulted in deceases in the proportion of OC on clay, and compensating increases in the proportions of OC on sand and silt fractions (Fig. 3). When the concentration of bulk soil OC rose to 20 to 30 g C kg^−1^, which is typical in the region for near surface (0–10 cm) Brookston clay loam under grain production, the proportion of OC in clay fraction dropped to 50–60%, while that in silt and sand fractions increased to 30–35% and 15%, respectively. With further increases in bulk soil OC up to 60 g C kg^−1^ soil, the proportion of OC associated with clay dropped to below 40%, while that in silt and sand fractions increased to about 30% (Fig. 2).

## Discussion

Segregating OC into several pools with different decay rates is common for soil organic carbon sequestration models^1^,^11^,^21^. The conceptual nature of these pools makes the individual pools only very loosely associated with measurable quantities obtained with existing analytical methods^11^. A commonly recognized and tested OC model that assumes OC pools associated with clay and silt is that of Hassink’s^5^, which has been used to estimate the capacity of soil to preserve C through attachment to silt and clay particles. The concept of soil capacity to store or sequester OC has been applied to many soils across the world^6^,^7^,^8^,^11^,^22^. However, the relationship between silt + clay content of soil and the amount of silt- and clay-protected soil C varied with different types of land use activities and clay type, as well as for different definitions of silt plus clay size class^11^. We eliminated the clay type (mineralogy) effect by studying one soil type (Brookston clay loam), and hence our relationships are primarily controlled by differences in carbon input or output induced by various management practices.

When bulk soil OC was <20 g C kg^−1^ soil, up to 98% of the OC was associated with the silt + clay size fraction, and when bulk soil OC was >30 g C kg^−1^ soil, 70–80% of the OC was associated with silt + clay. This indicates that the fine particle sizes (<53 μm) of Brookston clay loam play a key role in OC retention. In addition, the maximum soil OC associated with soil particles <53 μm diameter was much greater than the “protective capacity” or “saturation level” predicted by a recognized carbon storage model for Canadian soils (40 plus g C in clay + silt fraction kg^−1^ soil)^6^; and furthermore, the amount of OC on fine particles continued to increase with increasing bulk soil OC with no sign of plateau.

The slope, (incremental rate of C associated with clay plus silt particles with increase in clay pus silt particle contents), of the Carter *et al*.^6^ model (0.27) was comparable to the slope for the Hassink^5^ model (0.37), the Six *et al*.^11^ model (0.21–0.41), and the Feng *et al*.^8^ model (0.33 for 2:1 minerals). This was surprising, as the size criterion for fine particles differed substantially between the Carter *et al*.^6^ model (<53 μm) and the other models (<20 μm). For Brookston clay loam under cash crop management, the Carter *et al*.^6^ model predicts saturation levels of 22.5–33.7 g C kg^−1^ for silt + clay (Fig. 3). However, OC on silt + clay continued to increase to 40–45 g C kg^−1^ when bulk soil OC increased to 60–70 g C kg^−1^ due to long-term sod management (Fig. 3). It therefore seems that the OC storage/protective capacity of a soil can be determined by both the amount of fine particles and rate of C input, rather than solely by the amount of fine soil particles, as assumed by most carbon storage models.

Clay mineral surface area has been used in place of clay concentration to estimate the sequestration potential of soils. However, using sieved (<2 mm) topsoil and labelled organic carbon, Vogel *et al*.^20^ found that only some of the clay-sized surfaces can bind organic matter. Surprisingly, Vogel *et al*.^20^ noted that <19% of visible clay mineral surfaces accumulated labelled OC, and this OC was found to be preferentially associated with organo-mineral clusters with rough surfaces. Other studies have also found that organic carbon is unevenly distributed on clay surfaces, and does not cover the mineral surface as a monolayer^23^,^24^. Although different soil minerals have different specific surface areas which are positively correlated with OC in bulk soil and on soil fractions^13^,^25^,^26^, few studies have examined the OC holding capacity of soil minerals except Keil *et al*.^12^ and Mayer^13^ who proposed the monolayer-equivalent C loading of ~1 mg C m^−2^ as a potential maximal OC associated with fine particles based on data from marine sediments and upland soils. Soils with OC loading less than ~1 mg C m^−2^ are more likely to represent agricultural soils or deep soil horizons with low C inputs^24^,^27^; therefore, the concept of ~1 mg C m^−2^ of holding capacity may not be an accurate estimate of maximal OC sequestration. Our results support the view that only a limited portion of fine mineral surfaces contribute to OC sequestration and more work is needed to elucidate the impact of amount of carbon input on OC saturation levels.

The Brookston clay loams used in this study are poorly drained Dark Grey Gleysols with relatively uniform clay mineralogy and clay content down to 100 cm^28^. Our large range of soil OC concentrations reflects differences in sampling depth and agronomic management, including cropping, tillage, and organic amendment applications. We trust that this large range of soil OC concentrations for one soil type (Brookston clay loam) provided a unique opportunity to look at the relationship between OC “saturation” associated with fine particles (<53 μm) and the concentrations of OC in bulk soil. Empirical carbon sequestration models suggest that OC accumulation on fine soil particles reaches a maximum concentration; however, the concentrations of OC on the fine fraction of Brookston clay loam is far below the modelled “potential maximal levels”, particularly for the OC in the silt-sized fraction. The OC concentrations associated with fine particles in cropped Brookston clay loam (excluding sod) are about 20–25 g C kg^−1^ soil, which are substantially below the saturation levels of 77 g C kg^−1^ for OC predicted by the model (Table 2). These results may suggest that the so-called “protective capacity” or “the maximal level” of OC on fine particles of Brookston clay loam are actually a “steady state” equilibrium level which is much less than the total/absolute saturation level, and which is at least partially determined by the cropping system. This is consistent with the view of Torn^29^ that OC protection/sequestration varies with soil environment. Although we believe that the influence of soil texture on soil OC storage capacity was not as important as carbon input in this study, the authors acknowledge that soil texture could have been a greater factor if a wider range of soil textures were used in this study.

Using the “protective capacity” concept, Hassink^5^ proposed that if the amount of soil OC is below the “storage capacity” of the fine particles, adding organic matter will preferentially increase OC in the fine particles (silt plus clay), and Carter^2^ further proposed that this increase will continue until the fine particle “saturation level” is reached. Tsutsuki and Kuwatsuka^16^ and Carter *et al*.^6^ also found in their models that soil OC in coarse size fractions would increase only when soil OC in the clay plus silt sized fractions was at or near their predicted OC storage capacity. This appears consistent with our findings in that OC in the sand fraction of Brookston clay loam increased only after the OC concentration in bulk soil exceeded about ~25–30 g C kg^−1^. This level of soil OC (25–30 g C kg^−1^) is typical for the near-surface of cash-cropped Brookston clay loam in this study, and appears to be “steady state equilibrium” for the current management systems. In contrast to other studies, however, we found that not only sand OC, but also silt and clay OC, continued to increase with increasing bulk soil OC.

In summary, the ability of clay and silt to “store” OC appears to exist for Brookston clay loam soil, but the apparent OC storage capacity of the clay and silt fraction seems much greater than the capacity predicted by existing OS sequestration models. Although the OC on the sand fraction of Brookston clay loam showed a sharp increase after the OC on the clay fraction reached a certain level, the OC on clay and silt continued to increase at a reduced rate. These results support our hypothesis that literature estimates of OC storage capacity on clay and silt can be substantially underestimated. It is also appears that soil carbon sequestration is determined by more than the “protective capacity” of the silt plus clay particle sizes.

## Methods

The Brookston clay loam soil (Canadian Classification: Orthic Humic Gleysol; USDA Soil Taxonomy: fine, loamy, mixed mesic Typic Argiaquoll) at the field site (Eugene Whelan Research Farm, Agriculture and Agri-Food Canada, Woodslee, Ontario, lat. 42°13′N, long. 82°44′W) is a poorly drained lacustrine soil with an average plow layer OC concentration of 20.8 g C kg^−1^, and average plow layer texture of 265 g kg^−1^ sand, 327 g kg^−1^ silt, and 408 g kg^−1^ clay. This is why most Brookston clay loam soil in these studies and in southwestern Ontario are systematically tile drained. The Brookston clay loam soils comprise about 80% of the agricultural land in Southwestern, Ontario, Canada. Soil samples were collected from three long-term field experiments, including: i) an organic amendment study which received one-time applications (fall 1997) of household food waste compost (75 Mg ha^−1^, 150 Mg ha^−1^, 300 Mg ha^−1^), yard waste compost (75 Mg ha^−1^), and pig manure plus wheat straw compost (75 Mg ha^−1^) (see Yang *et al*.^30^ and Reynolds *et al*.^31^ for details); ii) a long-term fertilization and rotation experiment (initiated 1959) consisting of three cropping treatments (continuous corn, corn-oat-alfalfa-alfalfa rotation, continuous bluegrass sod) and two fertilizer treatments (fertilized, not fertilized) with each phase of the rotation present every year (see Drury *et al*.^32^ for details); and iii) a long-term tillage study (initiated in 1983 and modified in 1996) with corn under moldboard plow tillage, ridge tillage and no-tillage, plus continuous Kentucky bluegrass sod (see Yang *et al*.^33^ for details).

Soil samples were collected 10 years after initiation of the organic amendment study, 50 years after initiation of the fertilization and crop rotation study, and 24 years after initiation of the tillage study. A total of 221 soil cores (3.3 cm diameter) were obtained using a tractor-mounted Concord soil sampler, fitted with polycarbonate tube inserts. The soil cores were cut into six segments (0–5, 5–10, 10–20, 20–30, 30–40, and 40–60 cm) for the organic amendment and fertilization-rotation studies, and into five segments (0–5, 5–10, 10–20, 20–30, 30–40 cm) for the tillage study. Replicate samples from the same depth were pooled within each experiment and visible crop residues removed. The pooled samples were passed through a 2 mm sieve and air-dried, and then separated into the clay (<2 μm), clay + silt (<53 μm), and sand (53–2000 μm) particle size fractions using sonication^34^. Sonication was performed on a 20 g soil suspension (oven-dry equivalent) with 80 mL distilled water (1:4 mass ratio) in a 250-mL glass beaker. The sonication probe tip was inserted 17 mm below the suspension surface. Three replicate suspensions were analyzed for each soil sample. The samples were sonicated with an energy level of 750 J mL^−1^ soil suspension. The concentrations of OC in bulk soil, and in the sand, clay, and silt + clay fractions were determined using a CN 2000 analyzer (LECO Corporation, MI, USA). Because the soil is free of carbonates, the total C concentration is equivalent to the soil OC concentration. The OC in the silt fraction was calculated by weight proportion using^34^:


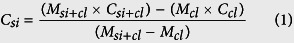


where *M*_*si+cl*_ is weight percentage (%) of the silt + clay fraction, *M*_*cl*_ is weight percentage of the clay fraction, and *C*_*si*_, *C*_*cl*_ and *C*_*si+cl*_ are the organic carbon concentrations (g C kg^−1^ soil) in the silt, clay, and silt + clay fractions, respectively.

Relationships among OC concentrations on different soil particle sizes and bulk soil were characterized using SigmaPlot V12.0 (SSI, San Jose, California) to (best) curve-fit various single-pool growth models to OC data. Since the OC in sand size particles grew exponentially with no apparent maximum, equation (2) was used for the sand model:


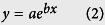


where *y* (g kg^−1^ soil) and *a* (g kg^−1^ soil) are, respectively, the current and antecedent concentrations of OC on sand-size particles, *b* (kg soil g^−1^) is a constant describing the rate of accumulation/depletion of OC, and *x* (g kg^−1^ soil) is the concentration of OC in bulk soil. For silt and clay, an exponential growth to maximum model produced the best fits:


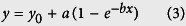


where *y* (g kg^−1^ soil) and *y*_*0*_ (g kg^−1^ soil) are, respectively, the current and antecedent OC concentrations on silt or clay, (*y*_*0*_ + *a*) (g kg^−1^ soil) is the storage capacity or saturation level for OC, *b* (kg soil g^−1^) is a constant describing the rate of accumulation/depletion of OC, and *x* (g kg^−1^ soil) is the concentration of OC in bulk soil. Relationships among the proportional changes of OC in particle size fractions as related to OC content in bulk soil were also evaluated using best empirical curves (Table 3).

## Additional Information

**How to cite this article**: Yang, X.M. *et al*. How do changes in bulk soil organic carbon content affect carbon concentrations in individual soil particle fractions? *Sci. Rep*. **6**, 27173; doi: 10.1038/srep27173 (2016).

## Figures and Tables

**Table 1:** Selected properties of bulk soil and soil particle size fractions (sand, silt, clay).

	Bulk soil	Particle size distribution			Carbon concentrations, particle size basis			Carbon concentrations, bulk soil basis		
	OC*	Sand*	Silt*	Clay*	Sand	Silt	Clay	Sand	Silt	Clay
	(g kg^−1^)	(g kg^−1^ bulk soil)			(g C kg^−1^ particle)			(g C kg^−1^ bulk soil)		
Max	70.8	423	407	495	66.9	64.4	84.0	28.3	20.7	28.9
Min	4.7	200	261	236	0.4	1.9	6.2	0.1	0.6	2.9
Mean	20.8	265	327	408	6.4	21.3	29.9	1.8	7.1	11.8
Median	21.4	256	331	404	4.0	22.1	31.8	1.0	7.5	12.9
s.d. (n=271)	9.9	32.2	22.5	38.5	8.2	11.1	12.4	2.8	3.9	4.2
CV (%)	47.4	12.1	6.9	9.4	128	52.1	41.4	151	54.4	35.8

^*^OC, organic carbon; Sand, 53–2000 μm; Silt, 2–53 μm; Clay, <2 μm.

**Table 2:** Model constants (*a, b, y*_*0*_), coefficients of determination (*R*^2^) and probability value (*P*) for relationships between OC concentration in particle size fractions (*y*, g C kg^−1^ soil particle) and OC concentration in bulk soil (*x*, g C kg^−1^ soil).

	Sand (53–2000 μm)	Silt (2–53 μm)	Clay (<2 μm)
	OC	OC	OC
	*y* = *a* × *e*^*bx*^	*y* = *y*_*0*_ + (*a1 − e*^*-bx*^)	
*y*_*0*_	–	−3.113	0
*a* (*g kg*^*−1*^)	2.075	205.4	118.3
*b* (*g*^*−1 *^*kg*)	0.051	0.006	0.014
*R*^2^	0.730	0.964	0.948
*P* value	<0.0001	<0.0001	<0.0001

**Table 3:** Model constants (*a, b*), coefficients of determination (*R* ^2^) and probability value (*P*) for the proportional changes of organic carbon concentration (OC) in particle size fractions, *y* (%), as related to OC in bulk soil, *x* (g C kg^−1^ bulk soil).

	Carbon in	Carbon in	Carbon in
	Sand (53–2000 μm)	Silt (2–53 μm)	Clay (<2 μm)
	*y* = *a* × *e*^*bx*^	*y* = *a*(*1 − e*^*−bx*^)	*y* = *a* ×* e*^*−bx*^
*a* (%)	2.75	35.7	80.4
*b* (g^−1 ^kg)	0.040	0.163	0.014
*R*^2^	0.620	0.444	0.734
*P* value	<0.0001	<0.0001	<0.0001
